# Post-Marketing Safety of Spinal Muscular Atrophy Therapies: Analysis of Spontaneous Adverse Drug Reactions from EudraVigilance

**DOI:** 10.3390/jcm14093173

**Published:** 2025-05-03

**Authors:** Andrej Belančić, Petar Mas, Lara Miletić, Barbara Kovačić Bytyqi, Dinko Vitezić

**Affiliations:** 1 Department of Basic and Clinical Pharmacology and Toxicology, Faculty of Medicine, University of Rijeka, 51000 Rijeka, Croatia; dinko.vitezic@medri.uniri.hr; 2 Agency for Medicinal Products and Medical Devices, 10000 Zagreb, Croatia; petar.mas@halmed.hr (P.M.); lara.miletic@halmed.hr (L.M.)

**Keywords:** spinal muscular atrophy, pharmacovigilance, adverse drug reactions, drug safety, nusinersen, onasemnogene abeparvovec, risdiplam

## Abstract

**Background/Objectives**: Spinal muscular atrophy (SMA) treatment has evolved with the approval of nusinersen, onasemnogene abeparvovec, and risdiplam. This study aims to assess the post-marketing safety profile of these therapies through the spontaneous adverse drug reaction (ADR) reports available in EudraVigilance (EV). **Methods**: Data from EV were retrieved via adrreports.eu for the suspected ADRs associated with nusinersen, onasemnogene abeparvovec, and risdiplam from their approval in the European Economic Area (EEA) to 31 December 2024. The ADR reports were exported and analysed using descriptive statistics in Microsoft Excel. Reporting odds ratios (RORs) with 95% confidence intervals (CIs) were calculated for suspected ADRs, focusing on reactions with a lower limit of the 95% CI exceeding 1. **Results**: A total of 3196, 806, and 956 individual case safety reports (ICSRs) were identified for nusinersen, onasemnogene abeparvovec, and risdiplam, respectively. The most frequently reported ADRs with significantly increased RORs included post-lumbar puncture syndrome (nusinersen: 11%), pyrexia (onasemnogene abeparvovec: 23%), and pneumonia (risdiplam: 9%). While some ADRs were therapy-specific, others were consistent with SMA disease progression and complications. Onasemnogene abeparvovec showed a notable prevalence of hepatotoxicity, while risdiplam was associated with gastrointestinal and respiratory events. **Conclusions**: To conclude, the analysis reinforces the known safety profiles of these SMA treatments while highlighting potential areas for further investigation. ADRs related to SMA complications require careful differentiation from true drug-related effects. Future pharmacovigilance efforts should focus on long-term safety assessments and real-world evidence to optimize treatment strategies.

## 1. Introduction

Spinal muscular atrophy (SMA) is a rare autosomal recessive neuromuscular disorder caused by homozygous deletions or mutations in the survival motor neuron 1 (SMN1) gene, leading to deficient levels of the SMN protein. This deficiency results in the progressive degeneration of lower motor neurons, manifesting as symmetrical muscle weakness and atrophy [1,2]. The clinical presentation of SMA is highly heterogeneous, ranging from severe neonatal forms with early mortality (Type 0) to adult-onset cases with mild proximal weakness (Type 4) [3,4].

The therapeutic landscape for SMA has transformed with the introduction of disease-modifying treatments (DMTs), which have significantly improved survival and motor function [5,6]. Nusinersen, an antisense oligonucleotide administered intrathecally, was the first approved therapy, receiving European Medicines Agency (EMA) authorization in May 2017 following positive outcomes from the ENDEAR and CHERISH trials [7,8,9]. Onasemnogene abeparvovec, a gene replacement therapy administered intravenously, was granted conditional EMA approval in May 2020, later converted to full authorization in May 2022, based on findings from the START and STR1VE trials [10,11,12,13]. Risdiplam, an oral splicing modifier, received EMA approval in March 2021 after demonstrating efficacy in the FIREFISH and SUNFISH trials [14,15,16]. These therapies, while revolutionary, necessitate rigorous long-term safety monitoring.

As their clinical use expands, ensuring the long-term safety of these therapies in real-world settings (in naïve patients as well as in patients who switched treatment) remains paramount. Post-marketing pharmacovigilance is critical to distinguishing true adverse drug reactions (ADRs) from complications inherent in SMA progression. This study aims to evaluate the post-marketing safety profile of nusinersen, onasemnogene abeparvovec, and risdiplam through spontaneous ADR reports from EudraVigilance (EV). By systematically analysing the reported safety signals, this research provides valuable insights into the treatment-associated risks, aiding clinicians, regulatory agencies, and policymakers in optimizing SMA management. In an era of rapidly advancing therapeutics, the pharmacovigilance community must act as the vigilant ‘bodyguard of drug safety’ [17], ensuring that the benefits of these novel therapies continue to outweigh their risks.

### Sneak Peek at the Pharmacovigilance Terminology and Methodology

EudraVigilance (EV) is the system for managing and analysing information on suspected adverse reactions to medicines, which have been authorised or are being studied in clinical trials in the European Economic Area (EEA). The European Medicines Agency (EMA) operates the system on behalf of the European Union (EU) medicines regulatory network. EV has been in operation since December 2001 [18,19].

Data are being submitted to EV by either the national competent authorities (NCAs) from each EEA member state or the marketing authorisation holders (MAHs) of medicinal products authorised in any EEA member state. The NCAs are submitting both serious and non-serious suspected ADR reports for the medicinal products received in their respective countries, while the MAHs are submitting both serious and non-serious suspected ADR reports for their medicinal products received from EEA countries and only serious reports for their products received in non-EEA (third) countries. All parties involved (NCAs, MAHs and EMA) collaborate together in detection of possible duplicates of cases [18].

Adrreports.eu is a website launched and maintained by the EMA since 2012 to provide public access to reports of suspected adverse drug reactions. This portal allows any interested individual or organisation to access a selected subset of anonymised data on suspected ADRs for certain medicinal products from the EV database by using either an active substance name for all centrally or nationally authorised medicinal products in the EEA or the registered name of a medicinal product for all centrally authorised medicinal products in the EEA. Data obtained from the adrreports.eu web portal contains all the data on reported spontaneous suspected ADRs following the administration of certain medicinal products regardless of their causality assessment, meaning that without a more detailed assessment it cannot be ascertained whether reported suspected ADRs are related to or caused by the medicine [19,20,21].

The EudraVigilance data analysis system (EVDAS) is a web-based application developed by the EMA to support pharmacovigilance activities in the EU/EEA with the main focus on signal detection and the evaluation of ICSRs. The EVDAS includes a measure of disproportionality, which is the reporting odds ratio (ROR). According to the EMA policy on the access to EV data on medicinal products for human use, access to the EVDAS is granted to NCAs, the European Commission, EMA and MAHs [22,23].

The MedDRA (Medical Dictionary for Regulatory Activities) is the international medical terminology developed under the auspices of the International Council for Harmonisation of Technical Requirements for Pharmaceuticals for Human Use (ICH). It is used to code suspected adverse drug reactions (ADRs) in the EV database; however, it can also be used in various stages of the medicinal product regulatory lifecycle, such as coding adverse events during clinical trials, and to prepare a summary of product characteristics (SmPC). The MedDRA has a hierarchical structure, where the MedDRA’s preferred term (PT) usually presents a single medical concept for a symptom, sign or disease. Terms of a higher level, such as the MedDRA’s system organ classes (SOC), can be used to summarise different PTs by their aetiology, manifestation site or purpose [24].

## 2. Materials and Methods

The EV database was searched using publicly accessible portal adrreports.eu for all the individual case safety reports (ICSR) of spontaneously reported suspected ADRs for medicinal products authorised for the treatment of SMA received from the date of their authorisation in EEA up to 31 December 2024. To obtain these cases, the database was searched using the active substance names, nusinersen, risdiplam and onasemnogene abeparvovec.

According to the European public assessment report (EPAR) accessible through the EMA website www.ema.europa.eu (accessed on 1 February 2025), Spinraza (nusinersen) was first authorised in the EEA on 30 May 2017, followed by Zolgensma (onasemnogene abeparvovec) on 18 May 2020 and Evrysdi (risdiplam) on 26 March 2021 [9,13,16].

All reported spontaneous ICSRs were exported to Microsoft Excel using the line listing function for each separate active substance. The exported file contained information on EU unique case number, type of report, date of transmission of report to EV, qualification of reporter (healthcare professional or non-healthcare professional), country of report (EEA or non-EEA), data on literature reference (for ICSR identified in medicinal literature), data on patient age group, patient sex, information on reported suspected ADRs (MedDRA PT term, duration, outcome and seriousness criteria) and information on suspect and concomitant medication (name of medicinal product or active substance, indication, action taken with drug, duration of use, dose and route of administration).

Descriptive statistics were used to describe the characteristics of patients and reported suspected ADRs from exported line listings. Microsoft Excel was used to perform analyses of exported cases. Since one ICSR generally refers to a single patient, when analysing patient data and case characteristics the number of individual cases for each active substance was used as a denominator. It should, however, be noted that one ICSR can contain multiple reported suspected ADRs and, therefore, when analysing data on suspected ADRs, the total number of reported reactions was used as the denominator, which is higher than the total number of ICSRs.

The RORs were exported from the EVDAS for all spontaneous reported cases using the same search criteria applied as when extracting ICSRs from the adrreports.eu portal. The reporting odds ratios (RORs) with 95% confidence intervals (CIs) were used as the measure of disproportionality for suspected ADRs. The analysis focused on the most frequently reported ADRs of each medicinal product for which the lower limit of the 95% CI of the ROR exceeded 1.

Since the data presented and analysed in this paper consisted of only a publicly available subset of ICSR data elements, described as Level 1 access in the EudraVigilance data access policy, and no personal protected data that could identify an individual patient was disclosed, no access authorisation or approval from the ethics committee was needed [25].

## 3. Results

There were total of 3196, 806 and 956 individual cases with 7243, 3176 and 2156 adverse drug reactions reported for nusinersen, onasemnogene abeparvovec and risdiplam, respectively. The general details of the cases, such as the report year, country of occurrence, qualification of reporter, patient age and sex, and case seriousness, are presented in Table 1.

There were a total of 233, 148 and 77 PTs with at least three cases reported and for which the lower limit of the 95% CI of the ROR exceeded 1 for nusinersen, onasemnogene abeparvovec and risdiplam, respectively.

Table 2, Table 3 and Table 4 show the 15 most frequently reported PTs for which the lower limit of the 95% CI of the ROR exceeded 1 for the three medicinal products. For nusinersen, the three most frequently reported PTs were post-lumbar puncture syndrome, pneumonia and scoliosis (11%, 7% and 6% of reports, respectively). For onasemnogene abeparvovec, the three most frequently reported PTs, pyrexia, vomiting and aspartate aminotransferase, increased (23%, 20% and 18% of reports, respectively). For risdiplam, the three most frequently reported PTs were pneumonia, diarrhoea and death (9%, 8% and 4% of reports, respectively).

## 4. Discussion

Cumulatively, the highest number of ICSRs have been reported for nusinersen, which could have been expected since nusinersen was approved in the EU in May 2017 [9]; i.e., 3 years before onasemnogene abeparvovec (May 2020) [13] and 4 years before risdiplam (March 2021) [16]. Another explanation for the latter could also be the route of administration (e.g., intrathecal administration for nusinersen). The reporters were primarily healthcare professionals (77.2%, 89.2% and 68.1%, respectively). Since its approval, the number of ICSRs for onasemnogene abeparvovec has been stable (between 156 and 193 ICSRs per year). Significant decreases in the number of ICSRs per year has been identified for nusinersen, from around 500 ICSRs between 2018 and 2022 to 319 and 246 ICSRs in 2023 and 2024, respectively. On the other hand, the number of ICSRs per year reported for risdiplam has been increasing steadily since 2021. The observed decrease in ICSRs per year for nusinersen could be as a result of switching patients from nusinersen to risdiplam, since there were studies showing that risdiplam might even be a superior alternative to nusinersen [26], or at least that it is not inferior to nusinersen in regard to its effectiveness [27].

The patient age group was not specified in approximately one-third of cases. Slightly more than half of all cases (52.7%) reported for onasemnogene abeparvovec were reported for the age group 2 months–2 years, which is in line with the approved EU product information stating that there is limited experience with onasemnogene abeparvovec in patients 2 years of age and older. Only 6.9% of cases were reported in patients older than 2 years of age. Considering that the safety and efficacy of onasemnogene abeparvovec in these patients has not been established, it should not be used in patients older than 2 years of age. Nusinersen and risdiplam have cases reporting the patient age group from 0–1 month to more than 85 years old. The majority of cases were reported for age groups 3–11 years (17.5% and 12.6%, respectively), 12–17 years (15.6% and 7.5%, respectively) and 18–64 years (18.8% and 26.6%, respectively). The observed data indicate that nusinersen and risdiplam are primarily used in patients older than 2 years of age.

The ICSRs were more frequently reported for female than male patients for onasemnogene abeparvovec and risdiplam (42.2% vs. 37.2% and 50.7% vs. 37.7%, respectively). This could be in line with the findings that indicate that the female gender experiences a higher incidence of adverse drug reactions than the male gender [28]. The ICSR distribution per sex was almost equal for nusinersen (41.6% female vs. 43.3% male).

For nusinersen and onasemnogene abeparvovec, 51.2% and 53.0% of cases, respectively, were reported in non-EEA countries, while for risdiplam, 49.0% of cases were reported in non-EEA countries (thus 51.0% in EEA countries). It should be highlighted that all non-serious and serious ICSRs that were reported in EEA countries have to be sent to EV, but only the serious ICSRs from non-EEA countries. Since the data on non-EEA countries could be incomplete, i.e., it cannot be established whether all reported non-serious cases from non-EEA countries are available in EV, conclusions on the ICSRs distribution per region of origin (EEA vs. non-EEA) and per seriousness should not be made.

The analysis of the most frequently reported PTs with corresponding RORs (95% CI) for nusinersen identified already known adverse drug reactions as listed in the EU product information (vomiting and back pain) and the expected consequences of the method of administration, which is intrathecal use by lumbar puncture (PTs post-lumbar puncture syndrome and procedural pain). Several identified PTs are signs/symptoms of underlying disease rather than adverse drug reactions. Six out of fifteen identified PTs are related to respiratory system and respiratory tract infections (respiratory failure, respiratory disorder, respiratory tract infection, rhinovirus infection, pneumonia, and respiratory syncytial virus infection). Respiratory disorders are one of the major causes of morbidity and mortality in patients with Type I and II SMA [29]. Reduced chest wall and pulmonary compliance increase the mechanical load on the weak respiratory muscles, which could lead to the imbalance between load and capacity, and consequently to muscle fatigue and respiratory failure [30]. The primary respiratory complications in patients with Type II SMA include an ineffective cough with decreased airway clearance, nocturnal hypoventilation, diminished lung and chest wall development, and increased risk of pulmonary infection [29]. When patients have problems clearing airway mucus secretions it could lead to aspiration pneumonia, a frequent cause of death in this patient population [30]. Considering scoliosis is frequently diagnosed at an early age, with significant effects on the respiratory system [31], it is also most probably a sign/symptom of the underlying disease rather than ADR. The PT spinal muscular atrophy could be wrongly stated as ADR instead of indication or it could indicate that the drug was ineffective. Similarly, PTs death, fall and cardiac arrest could be related to the complications of underlying disease. However, for all these PTs, more in-depth analyses of cases should be performed before any conclusions can be made.

Among the 15 most frequently reported PTs for which the lower limit of the 95% CI of the RORs exceeded one for onasemnogene abeparvovec, 12 PTs are already listed in the EU product information, including pyrexia, vomiting, hepatotoxicity/transaminases increase, troponin I increase, and thrombocytopenia. In addition, the approved product information states that the need for close monitoring of liver function, platelet count and troponin-I after administration is to be considered when establishing the timing of onasemnogene abeparvovec treatment. Pneumonia, asthenia and decreased appetite are most likely complications of the underlying disease; however, this could be confirmed with more detailed analyses of available cases.

The most frequently reported PTs for risdiplam also included the already listed ADRs in the EU product information (diarrhoea and urinary tract infection) and disease symptoms/complications (pneumonia, respiratory failure, muscular weakness, respiratory disorder, COVID-19 and respiratory tract infection). Weight increase could in this population be observed as a benefit rather than an ADR since patients are frequently underweight as a result of bulbar dysfunction, dysphagia, and gastrointestinal dysmotility [32,33]. The PTs asthenia, sepsis and death could be related to the underlying disease or its complications rather than the risdiplam. Since most patients with Type I SMA use acid-reducing medication to improve gut motility, and use bowel-regulating agents [32], abdominal pain and constipation could also be related to the disease. A potential signal regarding the abnormal product taste is a quality concern and not safety. Nevertheless, more in-depth analyses could be performed for the PTs asthenia, sepsis, death, abdominal pain, constipation and the abnormal product taste. Continuing the exploration from an etiopathogenetic perspective is also highly important [34].

Given the high costs and complex safety profiles of SMA therapies, the present findings are highly relevant for payers and reimbursement decision-makers. Enhanced post-marketing safety monitoring and targeted patient selection strategies could optimize healthcare resource utilization while maximizing patient outcomes.

Considering that the analysis was performed on spontaneous cases from the European ADR database, it has several limitations. The dataset was limited to EEA countries, as data from non-EEA countries may be incomplete. Spontaneous reporting is highly dependent on the reporter’s motivation to report ADRs, so ADRs can be either underreported or overreported. The total number of patients treated with the drug is unknown; therefore, the ADR incidence cannot be calculated, nor can it be determined whether the reported medical conditions are indeed ADRs or complications of the underlying disease. Spontaneous reporting cases cannot be used to compare the safety profile of different treatments, but only to identify potential new safety signals and generate hypotheses for further research as shown in this analysis.

## 5. Conclusions

The post-marketing safety assessment of nusinersen, onasemnogene abeparvovec, and risdiplam through spontaneous ADR reporting provides valuable insights into their real-world safety profiles. Our findings reaffirm previously known ADRs and also suggest a need for the careful interpretation of reported reactions due to disease-related complications. The high number of post-lumbar puncture syndrome cases associated with nusinersen underscores the procedural risks associated with intrathecal administration. Similarly, onasemnogene abeparvovec’s hepatotoxicity profile aligns with regulatory warnings, emphasizing the necessity for liver function monitoring post-treatment. Risdiplam’s association with gastrointestinal and respiratory events, while expected, necessitates further evaluation, particularly concerning its long-term safety.

Future research should leverage real-world data registries and prospective cohort studies to enhance causality assessments and provide more robust long-term safety evidence. In fact, such data are already being collected, as all three drugs have mandatory post-authorization efficacy studies (PAESs), and the risk management plan (RMP) includes various studies to evaluate the safety concerns, such as long-term extensions of clinical trials and patient registry studies. Additionally, exploring biomarkers for the early detection of severe ADRs could refine risk mitigation strategies and improve patient outcomes. Continued pharmacovigilance efforts and collaborative global safety monitoring will be pivotal in optimizing the therapeutic approaches for SMA, ensuring both the efficacy and long-term safety for affected patients.

## Figures and Tables

**Table 1:** Characteristics of cases and patients for nusinersen, onasemnogene abeparvovec and risdiplam.

	Nusinersen		Onasemnogene Abeparvovec		Risdiplam	
Total Cases	3196		806		956	
	N	%	N	%	N	%
*Report year*						
2017	123	3.8%	N/A	N/A	N/A	N/A
2018	522	16.3%	N/A	N/A	N/A	N/A
2019	512	16.0%	21	2.6%	N/A	N/A
2020	484	15.1%	87	10.8%	19	2.0%
2021	486	15.2%	159	19.7%	135	14.1%
2022	504	15.8%	190	23.6%	214	22.4%
2023	319	10.0%	193	23.9%	308	32.2%
2024	246	7.7%	156	19.4%	280	29.3%
*Reporter country*						
EEA	1560	48.8%	379	47.0%	488	51.0%
Non-EEA	1636	51.2%	427	53.0%	468	49.0%
*Reporter qualification*						
Healthcare professional	2466	77.2%	719	89.2%	651	68.1%
Non-healthcare professional	730	22.8%	87	10.8%	305	31.9%
*Patient age group*						
0–1 month	25	0.8%	58	7.2%	4	0.4%
2 months–2 years	498	8.0%	425	52.7%	83	8.7%
3–11 years	601	17.5%	54	6.7%	120	12.6%
12–17 years	256	15.6%	1	0.1%	72	7.5%
18–64 years	560	18.8%	1	0.1%	254	26.6%
65–85 years	18	0.6%	0	0%	11	1.2%
More than 85 years	0	0%	0	0%	1	0.1%
Not specified	1238	38.7%	267	33.1%	411	43.0%
*Patient sex*						
Female	1330	41.6%	340	42.2%	485	50.7%
Male	1383	43.3%	300	37.2%	360	37.7%
Not specified	483	15.1%	166	20.6%	111	11.6%
*Serious report*						
Yes	2148	67.2%	672	83.4%	563	58.9%
No	1048	32.8%	134	16.6%	393	41.1%
*Seriousness criteria* *						
Death	387	12.1%	47	5.8%	114	11.9%
Life-threatening	60	1.9%	27	3.3%	15	1.6%
Hospitalization	1381	43.2%	262	32.5%	254	26.6%
Disabling	21	0.7%	5	0.6%	22	2.3%
Congenital anomaly	2	0.1%	0	0%	1	0.1%
Other	647	20.2%	492	61.0%	248	25.9%

* More than one seriousness criterion can apply to a serious case; therefore, the sum of all the seriousness criteria is greater than the total number of serious cases.

**Table 2:** Most frequently reported PTs with the corresponding RORs (95% CI) for nusinersen with the lower limit of the 95% CI ≥ 1.

MedDRA Preferred Term (PT)	N *	ROR (95% CI) **
Post-lumbar puncture syndrome	352	4932 (4211, 5777)
Pneumonia	236	5.8 (5.0, 6.6)
Scoliosis	182	371 (317, 433)
Procedural pain	133	75 (63, 89)
Vomiting	125	1.4 (1.1, 1.6)
Death	122	2.0 (1.6, 2.4)
Back pain	101	3.4 (2.8, 4.1)
Respiratory failure	75	8.3 (6.6, 10.5)
Respiratory tract infection	69	26 (20, 33)
Respiratory disorder	69	18 (14, 23)
Spinal muscular atrophy	61	3345 (2369, 4724)
Rhinovirus infection	58	216 (166, 283)
Fall	58	1.8 (1.4, 2.3)
Cardiac arrest	55	5.0 (3.8, 6.5)
Respiratory syncytial virus infection	48	47 (36, 63)

* Number of cases reporting the respective PT. ** ROR: reporting odds ratio; 95% CI: 95% confidence interval.

**Table 3:** Most frequently reported PTs with the corresponding RORs (95% CI) for onasemnogene abeparvovec with the lower limit of the 95% CI ≥ 1.

MedDRA Preferred Term (PT)	N *	ROR (95% CI) **
Pyrexia	189	4.3 (3.7, 5.1)
Vomiting	158	8.1 (6.8, 9.6)
Aspartate aminotransferase increased	143	64 (54, 77)
Thrombocytopenia	139	24 (20, 29)
Alanine aminotransferase increased	138	52 (43, 63)
Transaminases increased	96	62 (50, 77)
Hepatic enzyme increased	84	37 (30, 47)
Platelet count decreased	64	14 (11, 18)
Decreased appetite	57	6.4 (4.9, 8.4)
Troponin I increased	49	808 (601, 1086)
Liver function test increased	42	49 (36, 66)
Hypertransaminasaemia	40	88 (64, 121)
Pneumonia	39	3.7 (2.7, 5.0)
Asthenia	30	1.6 (1.1, 2.4)
Body temperature increased	28	7.3 (4.9, 11)

* Number of cases reporting the respective PT. ** ROR: reporting odds ratio; 95% CI: 95% confidence interval.

**Table 4:** Most frequently reported PTs with the corresponding RORs (95% CI) for risdiplam with the lower limit of the 95% CI ≥ 1.

MedDRA Preferred Term (PT)	N *	ROR (95% CI) **
Pneumonia	85	7.0 (5.6, 8.8)
Diarrhoea	81	3.0 (2.4, 3.7)
Death	43	2.3 (1.7, 3.2)
COVID-19	34	2.0 (1.4, 2.8)
Asthenia	32	1.5 (1.0, 2.1)
Abdominal pain	28	2.0 (1.4, 2.9)
Respiratory failure	22	8.1 (5.3, 12)
Constipation	18	2.9 (1.8, 4.6)
Abnormal product taste	17	39 (24, 63)
Muscular weakness	16	2.8 (1.7, 4.6)
Respiratory disorder	15	13 (8, 22)
Urinary tract infection	15	3.2 (1.9, 5.3)
Respiratory tract infection	14	17 (10, 29)
Sepsis	14	3.5 (2.1, 6.0)
Weight increased	14	2.0 (1.2, 3.3)

* Number of cases reporting the respective PT. ** ROR: reporting odds ratio; 95% CI: 95% confidence interval.

## Data Availability

Available upon reasonable request sent to the corresponding author.
